# Proteinuria as a Therapeutic Target in Advanced Chronic Kidney Disease: a Retrospective Multicenter Cohort Study

**DOI:** 10.1038/srep26539

**Published:** 2016-05-20

**Authors:** Chang-Hsu Chen, Hon-Yen Wu, Chieh-Li Wang, Feng-Jung Yang, Pei-Chen Wu, Szu-Chun Hung, Wei-Chih Kan, Chung-Wei Yang, Chih-Kang Chiang, Jenq-Wen Huang, Kuan-Yu Hung

**Affiliations:** 1 Division of Nephrology, Tungs’ Taichung MetroHarbor Hospital, Taichung City, Taiwan; 2 Division of Nephrology, Far Eastern Memorial Hospital, New Taipei City, Taiwan; 3 Division of Nephrology, Department of Internal Medicine, National Taiwan University College of Medicine and Hospital, Taipei City, Taiwan; 4 Division of Nephrology, National Taiwan University Hospital Yun-Lin Branch, Yunlin County, Taiwan; 5 Division of Nephrology, Da Chien General Hospital, Miaoli County, Taiwan; 6 Division of Nephrology, Buddhist Tzu Chi Hospital Taipei Branch, New Taipei City, Taiwan; 7 Department of Nephrology, Chi Mei Medical Center, Tainan City, Taiwan; 8 Division of Nephrology, National Taiwan University Hospital Hsin-Chu Branch, Hsinchu City, Taiwan

## Abstract

Current evidence of proteinuria reduction as a surrogate target in advanced chronic kidney disease (CKD) is incomplete due to lack of patient-pooled database. We retrospectively studied a multicenter cohort of 1891 patients who were enrolled in the nationwide multidisciplinary pre-end stage renal disease care program with a baseline glomerular filtration rate (GFR) <45 mL/min/1.73 m^2^ and followed longitudinally to investigate the effect of the change in proteinuria on renal death (defined as composite of dialysis and death occurring before initiation of dialysis). The group with a change in proteinuria ≤0.30 g/g (n = 1261) had lower cumulative probabilities of renal death (*p* < 0.001). In a linear regression model, a higher baseline proteinuria and a greater increase in proteinuria were associated with faster annual GFR decline. Cox’s analysis showed that every 1 unit increase in natural log(baseline proteinuria, 10 g/g) and every 0.1 g/g increase in the change in proteinuria resulted in 67% (HR = 1.67, 95% CI: 1.46–1.91) and 1% (HR = 1.01, 95% CI: 1.01–1.01) greater risk of renal death respectively after adjusting for the effects of the other covariates. Our study provided a patient-based evidence to support proteinuria as a therapeutic target in advanced CKD.

The rapidly increasing incidence and prevalence of chronic kidney disease (CKD) have posed serious problems for global public health^1^,^2^. The impact of CKD on the burden of health resources includes increased risk of mortality, end-stage renal disease (ESRD), cardiovascular disease, mineral and bone disease, and other comorbidities^2^. The incidence and prevalence of ESRD in Taiwan are among the highest in the world^3^. An annual report of the Bureau of National Health Insurance (BNHI) in 2007 showed that 7.2% of health-care expenditure in Taiwan was used to provide treatment for patients with ESRD, although these patients accounted for only 0.23% of the local population^4^. According to national surveillance in Taiwan, the total prevalence of CKD was 11.93%, which was much higher than that in United States^5^, but only 3.54% of patients were aware of having CKD^6^. A strategy that prevents those with CKD from progressing to ESRD is mandatory for reducing the burden of ESRD.

Since 2002, the publication of a clinical practice guideline on CKD^7^ by the National Kidney Foundation Kidney Disease Outcomes Quality Initiative (NKF KDOQI) has generated immense effects on research, clinical practice, and public health policy. In Taiwan, a nationwide CKD preventive program has been established with standard pre-ESRD multidisciplinary care for patients with stage 3b–5 CKD^4^. The program has been proven to be helpful in reducing the incidence of ESRD, mortality, and medical costs by means of a more effective diet and medical control according to the NKF KDOQI guidelines^4^,^8^,^9^.

Proteinuria is an independent risk factor for progressive kidney function decline as well as all-cause mortality^10^,^11^,^12^,^13^. Medical treatment that reduces proteinuria seems to be renoprotective against glomerular filtration rate (GFR) decline^14^,^15^,^16^,^17^,^18^,^19^. Besides, evidences showed that multimodal regimen targeting at proteinuria reduction could effectively retard the progression of CKD^20^,^21^. Proteinuria has been advocated as a potential target for treatment in CKD^22^,^23^,^24^. However, because of various definitions of proteinuria reduction and outcome variables, one systemic review addressed the necessity of evaluation of pooled individual patient-level database^25^. Besides, few studies included patients with stage 5 CKD^26^,^27^,^28^. By using a large population of patients with advanced CKD with multihospital collaboration in Taiwan, the present study investigated the hypothesis that proteinuria reduction benefits renal outcomes.

## Results

**Figure 1:**
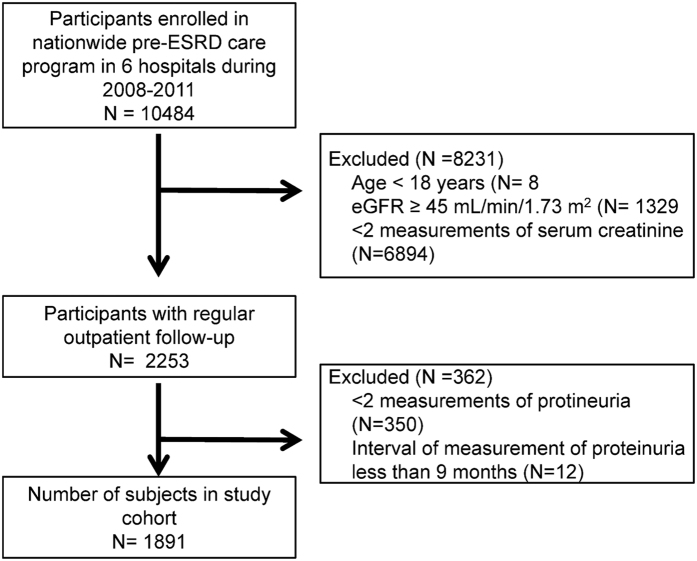
Overview of cohort formation.

**Figure 2:**
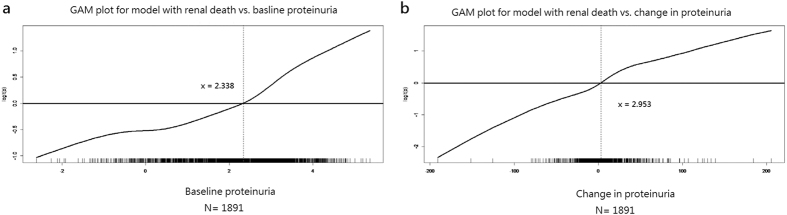
Generalized additive models for determination the cut-off points of (**a**) baseline proteinuria*; (**b**) change in proteinuria**. *The value of x-axis is transformed by natural log [10 × (baseline UPCR, g/g)]. **The value of x-axis is transformed by ΔUPCR (g/g) × 10. Note: logit(p) = natural log(p/1 − p), where p = probability of renal death. Abbreviations: UPCR, urine protein-to-creatinine ratio; ΔUPCR, change in UPCR.

The overview of cohort formation was shown in Fig. 1. Overall, 1891 participants with a mean age of 66 years and a mean baseline spot urine protein-to-creatinine ratio (UPCR) level of 1.63 ± 2.08 g/g were included in this study. The mean level of change in UPCR (ΔUPCR) was 0.26 ± 2.06 g/g and the mean annual GFR decline was −1.93 ± 5.89 mL/min/per 1.73 m^2^ per year. The patients with diabetic nephropathy had higher baseline UPCR compared to non-diabetic patients (2.92 ± 2.57 vs. 1.05 ± 1.48 g/g). By using generalized additive models (GAM), patients were stratified into a high proteinuria group (baseline UPCR >1.04 g/g) and a low proteinuria group (baseline UPCR ≤1.04 g/g) (Fig. 2a and Table 1). High proteinuria group had higher prevalence of diabetic nephropathy and hypertension and a lower baseline GFR. The mean age was younger, and this group exhibited faster GFR decline, higher mean arterial pressure (MAP).

A multiple linear regression analysis was applied for the independent determinants of annual GFR change (Table 2). Polycystic kidney disease, baseline proteinuria and the use of prescribed herbal medication are the three major factors that had negative associations with annual GFR change (i.e., the more negative value of annual GFR change, the more rapid decline in GFR). There are other conditions with negative association, such as increases in phosphate level and UPCR at follow-up (i.e., positive values of Δphospate and ΔUPCR). Conversely, increases in hemoglobin and a condition when changes in albumin were above 0.6 g/dl or below −0.2 g/dl are the two most important factors that had beneficial associations.

To investigate the difference in clinical characteristics of patients with CKD with different changes in UPCR, we divided all patients into two groups by ΔUPCR of 0.30 g/g after GAM analysis (Fig. 2b and Table 3). There was no significant difference in mean baseline UPCR, renin-angiotensin system (RAS) blockade, and interval between the two UPCR measurements. Patients in group of ΔUPCR >0.30 g/g had fewer men, younger mean age, more hypertension, lower baseline GFR, and higher proportion of diabetic nephropathy and stage 5 CKD. They also presented increased MAP and phosphate level at follow-up (i.e., more positive values of ΔMAP and Δphosphate). Besides, their annual GFR declined faster, their level of albumin decreased more (i.e., more negative values of Δalbumin), and greater proportion of them suffered from renal death.

**Figure 3:**
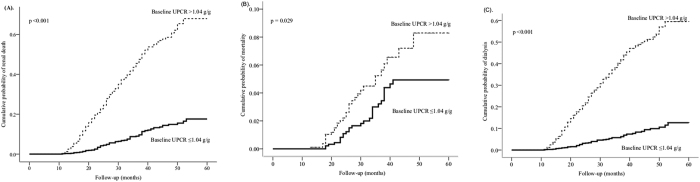
The Nelson–Aalen estimates for the cumulative hazard rates between the two groups stratified by baseline proteinuria (1.04 g/g). The patients with baseline UPCR ≥1.04 g/g had a higher hazard rate for composite renal death (**A**, *p* < 0.001), mortality (**B**, *p* = 0.029), and dialysis (**C**, *p* < 0.001).

**Figure 4:**
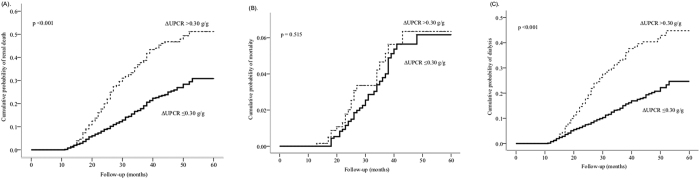
The Nelson–Aalen estimates for the cumulative hazard rates between the two groups stratified by change of proteinuria (ΔUPCR 0.3 g/g). The patients with ΔUPCR >0.3 g/g had a higher hazard rate for composite renal death (**A**, *p* < 0.001) and dialysis (**C,** *p* < 0.001). However, the mortality was not significantly different between the two groups (**B**, *p* =0.515).

In total, 367 participants (19.6%) suffered from renal death in a mean follow-up time of 32.0 ± 12.3 months. A Nelson-Aalen analysis revealed that the cumulative probabilities regarding renal death, mortality, and dialysis were lower in the low proteinuria group than those in the high proteinuria group (Fig. 3a–c). In another stratification with ΔUPCR, the cumulative probabilities of renal death and dialysis were also lower in the patients with ΔUPCR ≤0.30 g/g than those in the patients with ΔUPCR >0.30 g/g (Fig. 4a–c). However, there was no significant difference in terms of mortality between the two groups.

A Cox’s proportional hazards ratio analysis was used to determine the independent risk factors for renal death (Table 4). Every 1 unit increase in natural log(baseline proteinuria, 10 g/g) would result in a 67% increase in renal death risk (HR 1.67, 95% CI 1.46–1.91). Besides, every 0.1 g/g increases in ΔUPCR resulted in 1% greater risk of renal death (HR 1.01, 95% CI 1.01–1.01). The two edges of age distribution (<46 and >72 years) also increases the risk. Among 154 participants aged <46 years, only one died and 46 underwent dialysis. Of 661 participants aged >72 years, 45 died and 76 initiated dialysis. Phosphate levels of 3.7–7.2 mg/dL, body mass index (BMI) <23.9 or >36.4 kg/m^2^, and albumin levels of 3.2–4.4 g/dL also contributed to increments in odds ratio of renal death. Only a few participants had phosphate levels >7.2 mg/dL (*n* = 7), BMI >36.4 kg/m^2^ (*n* = 40), or albumin levels <3.2 g/dL (*n* = 32).

## Discussion

In this study of an advanced CKD population, we demonstrated that high baseline proteinuria (UPCR >1.04 g/g) was associated with rapid GFR decline and also predicted renal death. Additionally, less increase of proteinuria (ΔUPCR ≤0.30 g/g) was significantly associated with slower renal function decline and also predicted a lower probability of dialysis and mortality.

The Modification of Diet in Renal Disease (MDRD) study showed that high proteinuria >1.0 g/day was a predictor of renal progression^29^,^30^. By using GAM analysis, our study derived a consistent cut-off value to define a high-risk group in clinical practice. With the increment of baseline proteinuria, the rate of annual GFR decline and risk of renal death all increased. This is consistent with the previous literature that baseline proteinuria is almost linearly related to renal outcome^23^. Furthermore, one recent study showed that GFR decline was also strongly associated of the risk of ESRD and mortality^31^. In our study, baseline proteinuria is more strongly associated with renal progression and more predictive of renal death than GFR decline. One recent population-base cohort implied the similar concept by demonstrating that participants with heavy proteinuria but without overtly abnormal GFR had more rapid decline of kidney function than did those with moderately reduced GFR but mild proteinuria^32^.

Proteinuria reduction and residual proteinuria are predictors of renal disease progression and ESRD^18^,^23^,^33^,^34^,^35^. Short-term changes in proteinuria predicted GFR decline rate in non-diabetic nephropathy^34^. In type 2 diabetic nephropathy, a post hoc study showed that the hazard ratio gradually decreased if more reduction in percent change in proteinuria was achieved^23^. This effect was independent of treatment-related blood pressure changes^33^. The protective effects of proteinuria reduction persisted in late stage of CKD^26^,^28^,^36^,^37^. For example, one study in Chinese patients with creatinine clearance of 20 to 70 mL/min/1.73 m^2^ showed a significant correlation between the extent of proteinuria reduction and GFR decline^26^. However, only one of these studies enrolled stage 5 CKD^28^ and the ages of these study populations were younger than ours. Moreover, one study in non-diabetic nephropathy found that participants with higher residual proteinuria at 3 month had faster rate of GFR decline^34^. Similarly, residual proteinuria <0.5 g/day at 6 months had lowest hazard ratio of renal events in diabetic nephropathy^23^. Reducing proteinuria to <1 g/day or <0.5 g/day had been proposed as the goal of treatment^19^,^21^,^34^,^38^. Because of our study design, we were unable to demonstrate the effect of residual proteinuria. However, our study further extended the beneficial effect of proteinuria reduction to population of older and more advanced CKD.

Comparing to baseline proteinuria, the effects of changes in proteinuria were smaller on both GFR decline and renal death. Participants with higher baseline proteinuria reflected more severe degree of nephron dysfunction and damage^22^. In one study, although the low baseline proteinuria group had less proteinuria reduction and benefited less from the treatment, their renal outcome is better comparing to the overt proteinuria group^35^,^39^. Another study concluded that baseline proteinuria is the best independent predictor of disease progression and ESRD in non-diabetic proteinuric CKD^40^. However, a worsening proteinuria invariably predicts poor outcome^19^,^22^. Instead of precluding the benefit of proteinuria reduction, our finding supported the importance of early detection and referral of CKD before progression to overt proteinuria^41^.

In contrast to previous literature^42^,^43^, diabetes mellitus (DM) did not appear to be an independent risk factor in terms of GFR decline and renal death in our study. The effect of DM may have been embedded in some highly correlated factors, such as baseline UPCR and ΔUPCR, and became insignificant in multiple regression analysis. In addition, non-diabetic patients without using RAS blockade in our study were associated with better renal progression in the linear regression analysis. Recent meta-analyses had shown that RAS blockade was renoprotective in diabetic patients^44^,^45^, and KDIGO guideline^46^ also strongly recommended RAS blockade use in non-diabetic patients with CKD and severely increased proteinuria. Although less than a half of our participants received RAS blockade, we found that more patients were classified into ΔUPCR **≤**0.3 g/g group, which might inferred the effectiveness of multidiscipline care in controlling proteinuria. Besides, one study showed that still a substantial number of participants with diabetes and nephropathy treated with losartan had no reduction in proteinuria^33^. This may explain why our participants who could not achieve control of proteinuria had more often diabetic nephropathy. Use of RAS blockade in advanced CKD was considered safe in post hoc analyses of two randomized control trials and two trials in Chinese population^26^,^28^,^36^,^37^. However, the mean ages of these trials were younger than ours and current evidence of RAS blockade use in elderly CKD patients was still limited^47^. One recent study with a small number of elderly patients with CKD also showed that discontinuation of RAS blockade delayed the onset of dialysis^48^. Therefore, monitoring renal function is mandatory when using RAS blockade in patients with advanced CKD. Other modifiable risk factors including hemoglobulin, phosphate level, BMI, and blood pressure were also important in predicting renal death (see Supplementary Discussion). These results implied the goals and directions of multidisciplinary care in advanced CKD patients.

The strength of our study is that our participants received standard multidisciplinary care based on the NKF KDOQI guidelines instead of specific drug therapy, as proposed by previous literature^49^. Our cohort was older and we enrolled a substantial number of patients with stage 5 CKD. The mean annual GFR decline rate in our cohort was below most of the studies in CKD populations^46^, and two-thirds of our participants had stable to reduced proteinuria during study period. By using a patient-level pooled cohort with standardized care during a long period, as proposed by the previous literature^25^, our study provided relatively strong evidence for surrogacy.

There were several limitations in our study. First, due to retrospective study design, we were unable to adjust specific interventions and pharmacotherapy other than RAS blockade or to clarify the characteristics that lead to different degrees of proteinuria reduction under same treatment strategy. Second, our study population comprised exclusively of Taiwanese with advanced CKD who were referred to multi-discipline care. For examples, patients with more rapid progression who are unable to collect subsequent proteinuria were excluded. Selection bias might exist. Third, the MDRD equation was developed based on younger Caucasian subjects (aged 50.6 ± 12.7 years)^46^ and it may not correctly estimate the GFR among our patients. Moreover, one study showed that indirect formulas failed to provide reliable estimation of renal function changes over time^50^. Fourth, we used urine total protein instead of urine albumin, which is proposed by KDIGO guideline as a more sensitive marker^46^. However, urinary albumin and total protein perform equally in prediction of renal outcomes and mortality in patients with CKD^46^,^51^,^52^, and measuring urinary albumin is more costly than measuring total protein^46^,^51^. Besides, UPCR was significantly correlated with 24-hour urine protein excretion and highly predictive for disease progression^53^. Therefore, our program used UPCR as a marker during long-term surveillance. Fifth, patients with CKD may have diverse pattern of GFR trajectory^54^, and determination of renal progression by measuring two GFR in a time period may less accurately estimate the slope of GFR decline. Besides, serial measurement of concurrent time-varying proteinuria, GFR and associated clinical condition might be needed.

In summary, this multicenter retrospective study including participants with advanced CKD under multidisciplinary care in nephrology clinics showed that high proteinuria was associated with rapid GFR decline and also predicted renal death. The cut-off level of UPCR 1.04 g/g could be used in clinical practice to classify a high-risk group. Additionally, proteinuria reduction was significantly associated with slower renal function decline and also predicted risk of dialysis. To aim at improving renal outcome, the clinician can adjust the treatment policy to reduce proteinuria as much as possible. Our study supported the concept that proteinuria is a therapeutic target in patients with advanced CKD.

## Methods

### Study Design and Population

This is a retrospective cohort study conducted in six collaborative hospitals in Taiwan from 2008 to 2011. Patients who joined the nationwide pre-ESRD care program were enrolled. The GFR was estimated by using the MDRD study equation^7^: GFR = 186 × Serum Cre^−1.154^ × Age ^−0.203^ × (0.742 if female). We selected patients aged above 18 years with a baseline GFR <45 mL/min/per 1.73 m^2^ and at least two measurements of serum creatinine. We further excluded patients with less than two measurements of urine protein excretion (defined as spot urine protein-to-creatinine ratio, UPCR [g/g]). Patients with the interval of separate proteinuria measurements less than 9 months were also excluded. The severity of CKD was then staged based on the NKF KDOQI clinical practice guidelines^7^. The study was conducted in accordance with the Declaration of Helsinki and was approved by the medical ethics review boards of Tungs’ Taichung MetroHarbor Hospital, Far Eastern Memorial Hospital, National Taiwan University College of Medicine and Hospital and Da Chien General Hospital. Besides, waiver of the consent requirement was approved due to retrospective design and non-disclosure of patient- information.

### Pre-ESRD Care Program

The pre-ESRD care program in Taiwan included standardized interventions and multidisciplinary care that followed the NKF KDOQI guidelines^7^ and reimbursement policy of the BNHI. The members of this program included nephrologists, nurses, and dieticians, and integrated individual lectures focusing on nutrition, lifestyle, nephrotoxin avoidance, dietary principles, and pharmacological regimens as well as clinical evaluation and laboratory examinations were provided^4^,^8^. The criteria for dialysis initiation indicated by the BNHI included serum creatinine level ≥8 mg/dL or GFR <5 mL/min/per 1.73 m^2^; or serum creatinine level ≥6 mg/dL or GFR ≤15 mL/min/per 1.73 m^2^ with the presence of one or more uremic symptoms that threaten life or impair quality of life^8^,^9^.

### Patient Characteristics and Description of the Data Set

Demographics collected through medical records and registry data in each hospital included age; sex; employment status; education; smoking habits; drinking and betel nut use; original cause of renal failure; BMI; MAP; biochemical measurements including hemoglobulin, serum albumin, calcium, phosphate, and UPCR; comorbidity history; and antihypertensive drugs. Diabetic nephropathy was defined as UPCR ≥0.5 g/g in patients with diabetes mellitus without any other cause of renal failure.

### Outcome Assessment

Two primary outcomes were selected from our analysis. First, renal death was defined as the composite of initiation of dialysis and mortality (deaths occurring before initiating dialysis). Second, annual GFR change was estimated as the slope of a linear model including two GFR measurements, determined in mL/min/per 1.73 m^2^ per year. The change in covariates (Δ value) was defined as the value in follow-up minus baseline value of each parameter. A repeated-measures analysis was not used to assess changes during follow-up because the times of follow-up measurements varied across the patients.

### Statistical Analysis

A statistical analysis was performed using the IBM SPSS Statistics 21 software (IBM Corporation, Armonk, NY, United States) and the R 3.0.2 software (The R Foundation for Statistical Computing, Vienna, Austria). In statistical testing, a two-sided *p* value ≤ 0.05 was considered statistically significant. The distributional properties of continuous variables were expressed as mean (standard deviation, SD), categorical variables were presented by frequency and percentage, and the cumulative hazard rates of survival outcomes were estimated by the Nelson-Aalen method.

GAMs^55^ were fitted to determine the appropriate cut-off points of baseline UPCR and ΔUPCR for classifying patients into different groups. GAMs were fitted to detect nonlinear effects of continuous covariates and to identify appropriate cut-off point(s) for discretizing a continuous covariate, if necessary, during the stepwise variable selection procedure. Computationally, the vgam function (with the default values of smoothing parameters) of the VGAM package^56^,^57^ was used to fit GAMs for continuous and binary responses in R. Finally, the statistical tools of regression diagnostics for verification of proportional hazards assumption, residual analysis, detection of influential cases, and check of multicollinearity were applied to discover any model or data problems. The values of variance inflating factor (VIF) ≥10 in continuous covariates or ≥2.5 in categorical covariates indicate the occurrence of the multicollinearity problem among some of the covariates in the fitted regression model. If the required proportional hazards assumption was not satisfied in some covariates, the Cox’s proportional hazards model would be fitted to the long-form data with added interaction terms between survival time and the covariate(s) violating the proportional hazards assumption.

In univariate analysis, the two-sample *t* test, Wilcoxon rank-sum test, chi-square test, and Fisher’s exact test (if the expected values in any of the cells of a contingency table were <5) were used to examine the differences in the distributions of continuous variables and categorical variables within groups stratified by UPCR as well as ΔUPCR. Next, a multivariate analysis was conducted by fitting the linear regression model and Cox’s proportional hazards model to estimate the effects of risk factors, prognostic factors, or predictors on annual GFR change, occurrence of renal death, and time to renal death, respectively.

## Additional Information

**How to cite this article**: Chen, C.-H. *et al*. Proteinuria as a Therapeutic Target in Advanced Chronic Kidney Disease: a Retrospective Multicenter Cohort Study. *Sci. Rep.* **6**, 26539; doi: 10.1038/srep26539 (2016).

## Supplementary Material

Supplementary Information

## Figures and Tables

**Table 1:** General characteristics stratified by baseline proteinuria.

Variable	Baseline UPCR ≤1.04 g/g (*n* = 999)	Baseline UPCR >1.04 g/g (*n* = 892)	*P*value
Age (years)	68 ± 13	64 ± 13	<0.001
Men	634 (63.5)	442 (49.6)	<0.001
Married	767 (76.8)	661 (74.1)	0.177
Educated^*^	296 (29.6)	268 (30.0)	0.098
Employed	302 (30.2)	306 (34.3)	0.058
Smoker	189 (18.9)	190 (21.3)	0.197
Alcohol	92 (9.2)	76 (8.5)	0.60
Diabetic nephropathy	126 (12.6)	466 (52.2)	<0.001
Comorbidity			
Diabetes	397 (39.7)	518 (58.1)	<0.001
Cardiovascular disease	174 (17.4)	154 (17.3)	0.93
Hypertension	744 (74.5)	730 (81.8)	<0.001
PCKD	24 (2.4)	11 (1.2)	0.060
Herbal medication use	70 (7.0)	78 (8.7)	0.160
Prescribed	29 (2.9)	35 (3.9)	0.22
Non-prescribed	46 (4.6)	46 (5.2)	0.58
≥3 antihypertensive agents	108 (10.8)	148 (16.6)	<0.001
RAS blockade use	445 (44.5)	405 (45.4)	0.71
Baseline characteristics			
CKD stage 3b	419 (41.9)	171 (19.2)	<0.001
CKD stage 4	434 (43.4)	403 (45.2)	0.46
CKD stage 5	146 (14.6)	318 (35.7)	<0.001
MAP (mmHg)	93.8 ± 12.2	98.4 ± 13.5	<0.001
BMI (kg/m^2^)	25.5 ± 4.2	25.7 ± 4.6	0.39
Hemoglobin (g/dL)	11.6 ± 2.0	10.8 ± 2.0	<0.001
Corrected calcium (mg/dL)	9.0 ± 0.6	8.9 ± 0.6	0.011
Phosphate (mg/dL)	3.8 ± 0.8	4.2 ± 0.8	<0.001
Albumin (g/dL)	4.3 ± 0.4	4.1 ± 0.5	<0.001
UPCR (g/g)	0.42 ± 0.29	2.99 ± 2.36	<0.001
GFR (mL/min/per 1.73 m^2^)	27.24 ± 10.09	20.49 ± 9.88	<0.001
Changes			
ΔMAP (mmHg)	−0.5 ± 14.4	−1.8 ± 15.9	0.059
ΔBMI (kg/m^2^)	−0.2 ± 1.6	−0.3 ± 1.8	0.31
ΔHemoglobin (g/dL)	0.0 ± 1.4	−0.5 ± 1.6	<0.001
ΔCorrected Ca (mg/dL)	0.00 ± 0.6	0.0 ± 0.7	0.165
ΔPhosphate (mg/dL)	0.0 ± 0.8	0.4 ± 1.1	<0.001
ΔAlbumin (g/dL)	−0.1 ± 0.4	−0.1 ± 0.4	0.83
ΔUPCR (g/g)	0.36 ± 1.11	0.15 ± 2.76	0.029
Interval of changes (months)	19.1 ± 8.3	16.5 ± 6.0	<0.001
Annual GFR change (mL/min/per 1.73 m^2^ per year)	−0.07 ± 5.82	−4.01 ± 5.24	<0.001
Follow-up time (months)	34.6 ± 12.3	29.2 ± 11.7	<0.001
Composite renal death	88 (8.8)	279 (31.3)	<0.001
Mortality	28 (2.8)	32 (3.6)	0.33
Dialysis	60 (6.0)	247 (27.7)	<0.001

Abbreviations: UPCR, urine protein-to-creatinine ratio; PCKD, polycystic kidney disease; RAS, renin-angiotensin system; CKD, chronic kidney disease; MAP, mean arterial pressure; BMI, body mass index; GFR, glomerular filtration rate; Δ, changes in each covariate (defined as the level during follow-up minus the baseline level).

Note: Data are mean ± SD or frequency (%).

^*^Participant with at least junior high school education.

**Table 2:** Multiple linear regression analysis of the predictors associated with annual GFR change.

Covariate	Estimate ± standard error	*p* value
Intercept	1.38 ± 1.28	0.28
Baseline UPCR (per ln(10 g/g))	−1.59 ± 0.10	<0.001
ΔHemoglobin	0.94 ± 0.08	<0.001
ΔPhosphate	−1.23 ± 0.13	<0.001
Baseline GFR	−0.17 ± 0.01	<0.001
Baseline hemoglobin	0.60 ± 0.07	<0.001
Baseline phosphate	−0.78 ± 0.18	<0.001
ΔAlbumin (<−0.2 or >0.6 g/dL)	0.99 ± 0.24	<0.001
ΔUPCR (per 10 g/g)	−0.02 ± 0.01	<0.001
PCKD	−2.62 ± 0.84	0.002
Non-DM × Non-RAS blockade	0.71 ± 0.25	0.004
ΔBMI (<−0.4 or >2.4 kg/m^2^)	0.62 ± 0.23	0.007
Prescribed herbal medication use	−1.51 ± 0.62	0.015

*R*_2_ = 0.3183.

Abbreviations: GFR, glomerular filtration rate; UPCR, urine protein-to-creatinine ratio; Δ, changes in each covariate (defined as the level during follow-up minus the baseline level); PCKD, polycystic kidney disease; DM, diabetes mellitus; RAS, renin-angiotensin system; BMI, body mass index.

**Table 3:** Characteristics of participants stratified by level of change in proteinuria.

Variable	ΔUPCR ≤0.30 g/g (*n* = 1261)	ΔUPCR >0.30 g/g (*n* = 630)	*P* value
Age	67 ± 13	65 ± 13	0.025
Men	754 (59.8)	322 (51.1)	<0.001
Married	962 (76.3)	466 (74.0)	0.28
Educated^*^	392 (31.1)	172 (27.3)	0.098
Employed	407 (32.3)	201 (31.9)	0.88
Smoker	259 (20.5)	120 (19.0)	0.47
Diabetic nephropathy	365 (28.9)	227 (36.0)	0.002
Comorbidity			
Diabetes	592 (46.9)	323 (51.3)	0.079
Cardiovascular disease	226 (17.9)	102 (16.2)	0.37
Hypertension	959 (76.1)	515 (81.7)	0.005
PCKD	24 (1.9)	11 (1.7)	0.86
Herbal medication use	98 (7.8)	50 (7.9)	0.93
≥3 antihypertensive agents	158 (12.5)	98 (15.6)	0.075
RAS blockade use	566 (44.9)	284 (45.1)	0.96
Baseline characteristics			
CKD stage 3b	445 (35.3)	145 (23)	<0.001
CKD stage 4	548 (43.5)	289 (45.9)	0.33
CKD stage 5	268 (21.3)	196 (31.1)	<0.001
MAP (mmHg)	96.1 ± 13.1	95.6 ± 12.8	0.42
BMI (kg/m^2^)	25.5 ± 4.3	25.6 ± 4.5	0.67
Hemoglobin (g/dL)	11.4 ± 2.1	10.8 ± 2.0	<0.001
Corrected calcium (mg/dL)	9.2 ± 0.6	9.1 ± 0.6	0.095
Phosphate (mg/dL)	3.9 ± 0.8	4.1 ± 0.8	<0.001
Albumin (g/dL)	4.3 ± 0.4	4.2 ± 0.4	0.013
UPCR (g/g)	1.66 ± 2.29	1.57 ± 1.58	0.38
GFR (mL/min/per 1.73 m^2^)	25.10 ± 10.54	21.96 ± 10.24	<0.001
Changes			
ΔMAP (mmHg)	−2.6 ± 14.9	1.9 ± 15.0	<0.001
ΔBMI (kg/m^2^)	−0.2 ± 1.7	−0.2 ± 1.8	0.56
ΔHemoglobin (g/dL)	−0.2 ± 1.4	−0.1 ± 1.7	0.175
ΔCorrected calcium (mg/dL)	0.0 ± 0.6	−0.1 ± 0.7	0.092
ΔPhosphate (mg/dL)	0.1 ± 0.9	0.3 ± 1.2	0.002
ΔAlbumin (g/dL)	−0.1 ± 0.4	−0.2 ± 0.4	<0.001
ΔUPCR (g/g)	−0.55 ± 1.35	1.89 ± 2.27	<0.001
Interval of changes (months)	17.7 ± 7.0	18.2 ± 8.0	0.26
Annual GFR change (mL/min/per 1.73 m^2^ per year)	−1.58 ± 5.80	−2.62 ± 6.01	<0.001
Follow-up time (months)	32.5 ± 12.2	31.1 ± 12.6	0.015
Composite renal death	192 (15.2)	175 (27.8)	<0.001
Mortality	39 (3.1)	21 (3.3)	0.78
Dialysis	153 (12.1)	154 (24.4)	<0.001

Abbreviations: Δ, changes in each covariate (defined as the level during follow-up minus the baseline level); UPCR, urine protein-to-creatinine ratio; PCKD, polycystic kidney disease; RAS, renin-angiotensin system; MAP, mean arterial pressure; BMI, body mass index; GFR, glomerular filtration rate.

Note: Data are mean ± SD or frequency (%).

^*^Participant with at least junior high school education.

**Table 4:** Predictors of composite renal death by Cox’s model.

Covariate	Estimate ± se	Wald Chi-Square	Hazard ratio	95% Confidence interval	*p*value
Baseline hemoglobin	−0.088 ± 0.034	−0.265	0.92	0.86–0.98	0.087
Baseline UPCR* (per ln(10 g/g))	0.510 ± 0.069	7.436	1.67	1.46–1.91	<0.001
Baseline GFR	−0.273 ± 0.031	−8.858	0.76	0.72–0.81	<0.001
Baseline GFR × time to renal death	0.005 ± 0.001	4.575	1.00	1.00–1.01	<0.001
Annual GFR decline	−0.359 ± 0.047	−7.625	0.70	0.72–0.81	<0.001
Annual GFR decline × time to renal death	0.005 ± 0.002	3.181	1.01	1.00–1.01	0.002
ΔUPCR (per 10 g/g)	0.011 ± 0.002	5.577	1.01	1.01–1.01	<0.001
Men	0.518 ± 0.116	4.487	1.68	1.34–2.10	<0.001
Age (<46 or >72 years)	−0.471 ± 0.108	4.380	1.60	1.30–1.98	<0.001
Baseline BMI (<23.9 or >36.4 kg/m^2^)	0.397 ± 0.108	3.657	1.49	1.20–1.84	<0.001
Baseline phosphate (3.7–7.2 mg/dL)	0.429 ± 0.136	3.149	1.53	1.18–2.00	0.002
Baseline albumin (3.2–4.4 g/dL)	0.255 ± 0.118	2.169	1.29	1.02–1.63	0.030
Educated**	0.252 ± 0.121	2.090	1.29	1.02–1.63	0.037
Herbal medication use	0.308 ± 0.167	1.846	1.36	0.98–1.89	0.065

Goodness-of-fit assessment: Adjusted generalized *R*^2^ = 0.3669 > 0.15 and concordance = 0.89 > 0.7 (se = 0.016) indicated a very good fit.

Abbreviations: se, standard error; UPCR, urine protein-to-creatinine ratio; GFR, glomerular filtration rate; ΔUPCR, change in UPCR (defined as the level at follow-up minus the baseline level); BMI, body mass index.

*The value of baseline UPCR was natural log-transformed in regression analysis for making its distribution more symmetric.

**Educated = 1 for participant with at least junior high school education, 0 otherwise.
