# Supplementary material for: Proteinuria as a Therapeutic Target in Advanced Chronic Kidney Disease: a Retrospective Multicenter Cohort Study
Source: Sci Rep. 2016 May 20;6:26539. doi: 10.1038/srep26539 (PMC4873744; doi:10.1038/srep26539)
Supplement: Supplementary Information [file srep26539-s1.pdf]

Supplementary information

Proteinuria as a Therapeutic Target in Advanced Chronic Kidney Disease: a Retrospective Multicenter Cohort Study

Chang-Hsu Chen[1], Hon-Yen Wu[2], Chieh-Li Wang[3], Feng-Jung Yang[4], Pei-Chen Wu[5], Szu-Chun Hung[6], Wei-Chih Kan[7], Chung-Wei Yang[8], Chih-Kang Chiang[3], Jenq-Wen Huang[3], Kuan-Yu Hung[3]

[1]Division of Nephrology, Tungs' Taichung MetroHarbor Hospital, Taichung, Taiwan

[2]Division of Nephrology, Far Eastern Memorial Hospital, New Taipei, Taiwan

[3]Division of Nephrology, Department of Internal Medicine, National Taiwan University College of Medicine and Hospital, Taipei City, Taiwan

[4]Division of Nephrology, National Taiwan University Hospital Yun-Lin Branch, Yun-Lin County, Taiwan

[5]Division of Nephrology, Da Chien General Hospital, Miaoli, Taiwan

[6]Division of Nephrology, Buddhist Tzu Chi Hospital Taipei Branch, New Taipei, Taiwan

[7]Department of Nephrology, Chi Mei Medical Center, Tainan, Taiwan

[8]Division of Nephrology, National Taiwan University Hospital Hsin-Chu Branch, Hsin-Chu County, Taiwan

Supplementary Discussion

Definition of the change in proteinuria

There was no consensus on the definition of an early change in proteinuria[1]. Quartile decrease or percent decrease in proteinuria during a certain period of time was used by many studies[1-4]. One recent meta-analysis using both absolute and relative change in proteinuria concluded that proteinuria reduction from baseline to the first follow-up measurement between 2.5 and 13 months was associated with lower risk of the doubling of serum creatinine level, end-stage renal disease or death. The use of percent change by baseline albuminuria might be insufficient to separate the effects of the two correlated variables[5]. For example, one study showed that patients with higher baseline proteinuria benefits more from RAS blockade because of greater reduction in proteinuriain this group (relative risk of 0.2 per 1 g/day)[6]. This effect might be obscure if using percent changes. By using absolute changes in proteinuria, our study demonstrated that more proteinuria reduction resulted in slower GFR decline and lower risk of renal death[7].

Associated Risk Factors

Age predicted renal death but did not associate with the decline of GFR in the present study. The effects of age on GFR decline are still controversial[8,9].

The NKF KDOQI guideline had illustrated the conflicting results regarding age and progression rate in different studies[10]. Age <46 years as a risk factor might reflect a clinical fact that these younger patients with advanced CKD might have a more progressive underlying disease. Conversely, more participants >72 years died to reach the outcome of renal death than did those patients <46 years. This result echoed the result of a previous study that death is a competing risk for end stage renal disease in elderly patients with CKD[8]; therefore, the importance of multidisciplinary care is to decrease mortality in the aged group[11].

Baseline albumin levels of 3.2–4.4 g/dL and phosphate levels of 3.7–7.2 mg/dL predicted mortality in our study, and this finding was not consistent with the previous literature. A higher serum phosphorus level was associated with mortality in CKD[12], and hypoalbuminemia was a strong predictor of death in both, dialysis and non-dialysis CKD populations[13,14]. The small number of participants with baseline albumin levels <3.2 g/dL (n = 32) and phosphate levels >7.2 mg/dL (n = 7) would distort the effects of these two parameters. However, the protective effects of albumin level >4.4 g/dL and phosphate level <3.7 mg/dL would be in accordance with the aforementioned literature.

Herbal medicine use was also associated with faster renal progression.

Herbal medicine is popular in Taiwan and is associated with CKD risk[15]. We also demonstrated that herbal medicine use was a predictor of renal death in our study, and the KDIGO guideline advocated for not using herbal remedies in patients with CKD[16].

References

1 Stoycheff, N. *et al.* Early change in proteinuria as a surrogate outcome in kidney disease progression: a systematic review of previous analyses and creation of a patient-level pooled dataset. *Nephrol Dial Transplant* **26**, 848-857 (2011).

2 The GISEN Group (Gruppo Italiano di Studi Epidemiologici in Nefrologia). Randomised placebo-controlled trial of effect of ramipril on decline in glomerular filtration rate and risk of terminal renal failure in proteinuric, non-diabetic nephropathy. *Lancet* **349**, 1857-1863 (1997).

3 Brenner, B. M. *et al.* Effects of losartan on renal and cardiovascular outcomes in patients with type 2 diabetes and nephropathy. *N Engl J Med* **345**, 861-869 (2001).

4 Ruggenenti, P. *et al.* Renoprotective properties of ACE-inhibition in non-diabetic nephropathies with non-nephrotic proteinuria. *Lancet* **354**, 359-364 (1999).

5 Inker, L. A. *et al.* Early change in proteinuria as a surrogate end point for kidney disease progression: an individual patient meta-analysis. *Am J Kidney Dis* **64**, 74-85 (2014).

6 O'Seaghdha, C. M. & Fox, C. S. The fourth dimension: associations of

change in albuminuria over time. *J Am Soc Nephrol* **22**, 1186-1188 (2011).

7 Jafar, T. H. *et al.* Proteinuria as a modifiable risk factor for the progression of non-diabetic renal disease. *Kidney Int* **60**, 1131-1140 (2001).

8 O'Hare, A. M. *et al.* Age affects outcomes in chronic kidney disease. *J Am Soc Nephrol* **18**, 2758-2765 (2007).

9 Chen, S. C. *et al.* Slowing renal function decline in chronic kidney disease patients after nephrology referral. *Nephrology (Carlton)* **13**, 730-736 (2008).

10 National Kidney, F. K/DOQI clinical practice guidelines for chronic kidney disease: evaluation, classification, and stratification. *Am J Kidney Dis* **39**, S1-266 (2002).

11 Chen, Y. R. *et al.* Effectiveness of multidisciplinary care for chronic kidney disease in Taiwan: a 3-year prospective cohort study. *Nephrol Dial Transplant* **28**, 671-682 (2013).

12 Palmer, S. C. *et al.* Serum levels of phosphorus, parathyroid hormone, and calcium and risks of death and cardiovascular disease in individuals with chronic kidney disease: a systematic review and

meta-analysis. *JAMA* **305**, 1119-1127 (2011).

13 Owen, W. F., Jr., Lew, N. L., Liu, Y., Lowrie, E. G. & Lazarus, J. M. The urea reduction ratio and serum albumin concentration as predictors of mortality in patients undergoing hemodialysis. *N Engl J Med* **329**, 1001-1006 (1993).

14 Soriano, S., Gonzalez, L., Martin-Malo, A., Rodriguez, M. & Aljama, P. C-reactive protein and low albumin are predictors of morbidity and cardiovascular events in chronic kidney disease (CKD) 3-5 patients. *Clin Nephrol* **67**, 352-357 (2007).

15 Hsieh, C. F. *et al.* Increased risk of chronic kidney disease among users of non-prescribed Chinese herbal medicine in Taiwan. *Prev Med* **55**, 155-159 (2012).

16 Kidney Disease: Improving Global Outcomes (KDIGO) CKD Work Group. KDIGO 2012 Clinical Practice Guideline for the Evaluation and Management of Chronic Kidney Disease. *kidney int suppl* **3**, 1-150 (2013).
